# Tissue concentration of paraquat on day 32 after intoxication and failed bridge to transplantation by extracorporeal membrane oxygenation therapy

**DOI:** 10.1186/2050-6511-14-45

**Published:** 2013-09-06

**Authors:** Anna Bertram, Sascha Sebastian Haenel, Johannes Hadem, Marius M Hoeper, Jens Gottlieb, Gregor Warnecke, Stanislav Kaschinski, Carsten Hafer, W Nikolaus Kühn-Velten, Detlef Günther, Jan T Kielstein

**Affiliations:** 1 Department of Nephrology, Hannover Medical School, Carl Neuberg Street 1, Hannover 30625, Germany; 2 Department of Gastroenterology, Hepatology and Endocrinology, Hannover Medical School, Carl Neuberg Street 1, Hannover 30625, Germany; 3 Department of Pneumology, Hannover Medical School, Carl Neuberg Street 1, Hannover 30625, Germany; 4 Department of Cardiothoracic, Transplantation and Vascular Surgery, Hannover Medical School, Carl Neuberg Street 1, Hannover 30625, Germany; 5 Institute for Radiology, Hannover Medical School, Carl Neuberg Street 1, Hannover 30625, Germany; 6 Medical Laboratory of Bremen, Haferwende 12, Bremen 28357, Germany; 7 Hannover Medical School, Institute for Forensic Medicine, Carl Neuberg Street 1, Hannover 30625, Germany

**Keywords:** Paraquat, Poisoning, Extracorporeal membrane oxygenation, Lung transplantation

## Abstract

**Background:**

Paraquat is a highly toxic herbicide, which not only leads to acute organ damage, but also to pulmonary fibrosis. There are only anecdotal reports of rescue lung transplantation, as paraquat is stored and only slowly released from different tissues. Bridging the time to complete depletion of paraquat from the body could render this exceptional therapy strategy possible, but not much is known on the time interval after which transplantation can safely be performed.

**Case presentation:**

We report on a case of accidental paraquat poisoning in a 23 years old Caucasian man, who developed respiratory failure due to pulmonary fibrosis. The patient was listed for high urgency lung transplantion, and extracorporeal membrane oxygenation was implemented to bridge the time to transplantation. The patient died 32 days after paraquat ingestion, before a suitable donor organ was found. In postmortem tissue specimen, no paraquat was detectable anymore.

**Conclusion:**

This case report indicates that complete elimination of paraquat after oral ingestion of a lethal dose is achievable. The determined time frame for this complete elimination might be relevant for patients, in which lung transplantation is considered.

## Background

Paraquat (1,1’-dimethyl-4,4’ bipyridinium dichloride) is a highly toxic contact herbicide, which has been banned in Germany since 2007. However, there are still rare cases of paraquat intoxications with leftovers of paraquat purchased prior to the ban, mostly in form of suicide attempts. After oral ingestion, the intestinal absorption of paraquat is poor (1-5%), but still sufficient to elicit a severe and potentially fatal intoxication. Early toxicity includes oral, pharyngeal and gastrointestinal ulcerations and necroses, acute kidney injury and liver failure [1]. Ingestion of two or more mouthful of paraquat usually leads to circulatory failure within two days, whereas patients, who swallow not more than one mouthful, often survive the early phase of paraquat intoxication [2]. Late toxicity is mostly due to the storage of paraquat in alveolar macrophages, where it reacts with the highly abundant oxygen to form radicals and reactive oxygen species. The reactions of paraquat simultaneously deplete the body of antioxidants [3]. The consequences are pulmonary inflammation and fibrosis, which is the main cause of death in patients surviving the early phase of paraquat poisoning. In case of attempted suicide, the psychiatric background does not allow lung transplantation. In accidental paraquat ingestion, however, lung transplantation could be the only therapeutic strategy, if it is performed after complete depletion of paraquat from the body to prevent recurrence of fibrosis in the allograft. The first attempt of lung transplantation in paraquat intoxication in 1968 failed as the single lung transplantation was performed as early as 7 days after intoxication of a 15 years old boy [4]. Due to recurrence of fibrosis in the allograft, these authors speculated already that paraquat stored in the tissue caused fibrosis of the allograft. Hence, complete depletion of paraquat from the body to prevent fibrosis of the allograft due to redistribution of paraquat from the tissue is necessary. After what time this complete depletion would occur is unknown. Yet the first successful lung transplantation reported by Walder et al. [5] was performed 44 days after intoxication. Here, we report on a case of accidental paraquat intoxication, in which we attempted to bridge the time to transplantation with extracorporeal membrane oxygenation.

## Case presentation

**Figure 1:**
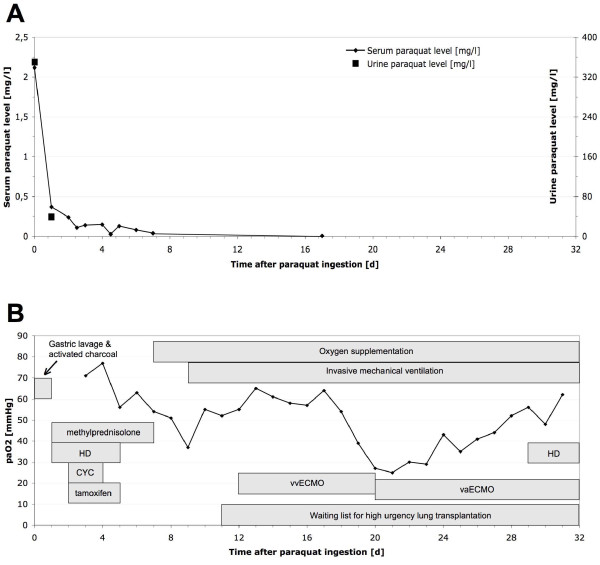
**Clinical course. A)** Paraquat serum and urine levels over time. Serum levels exceeding 0.6 mg/l at 6 h or 0.1 mg/l at 24 h predict an unfavourable outcome [6]. The low serum values on day 3 and 4 reflect post-dialysis values. **B)** Arterial partial oxygen tension and flow chart of therapeutic interventions (HD: hemodialysis; CYC: cyclophosphamide).

**Figure 2:**
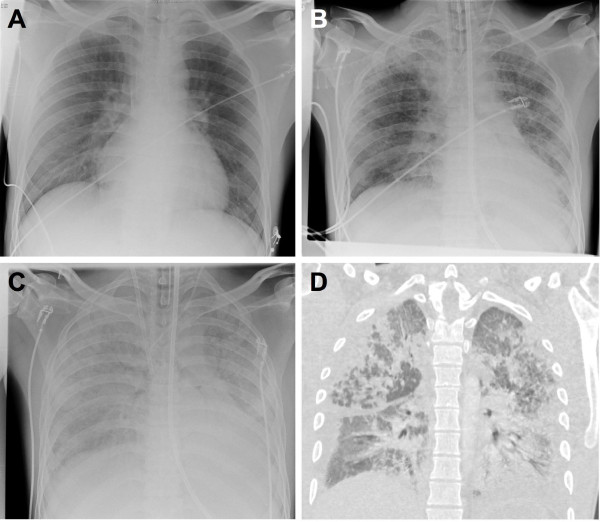
**Progressive pulmonary fibrosis.** Chest X-ray **(A-C)** and computed tomography **(D**; 11th day**)** demonstrating progressive pulmonary fibrosis on day 3 **(A)**, 9 **(B)**, and 12 **(C)**.

A 23 years old Caucasian man was admitted to a local hospital after reporting that he had accidentally ingested paraquat. Allegedly he had filled the paraquat (Gramoxone®, 20% [weight/volume]) into a soda bottle a few days before in order to facilitate the dosing of the herbicide at his job as a gardener, avoiding the use of the industrial size original container. On a Monday morning he confused the bottle with the paraquat aliquot with a real soda bottle and accidentally swallowed one mouthful, which probably accounts for an ingested amount of paraquat of 6–10 g. He immediately provoked emesis and presented to the nearest emergency room. There, a gastric lavage was performed, 40 g of activated charcoal were administered and paraquat levels in serum (2.12 mg/l) and urine (350.00 mg/l) were determined (Figure 1A), suggesting that the ingested amount of paraquat was at a lethal dose [6]. After developing acute non-oliguric kidney injury within 48 h, which is another known sign indicating a fatal outcome [7], the patient was transferred to our tertiary care centre for further treatment. At this time, the patient did not report any shortness of breath, nausea, pain or impaired diuresis. Physical examination revealed mild jaundice and swelling and redness of the throat. Laryngoscopy showed redness and necroses of the hypopharynx, the epiglottis and the vocal cords. Otherwise, physical examination was unremarkable, with normal findings for chest and abdominal examination and a normal chest X-ray (Figure 2A). Forced vital capacity was reduced to 38% predicted on day 4, the alveolar-arterial oxygen gradient was 34 mmHg. Laboratory values revealed pathological liver function tests, mildly elevated inflammatory parameters and acute kidney injury (Table 1), so daily hemodialysis was started. Using the GENIUS® system with a sleddFlux dialysator (Fresenius Medical Care GmbH, Bad Homburg, Germany) and a blood flow of 300 ml/min, a paraquat clearance of 122 ml/min was achieved. No paraquat (i.e., < 0.01 mg/l) was detectable in the collected spent dialysate. In addition, a therapy with methylprednisolone (5 × 1 g i.v.) and cyclophosphamide (15 mg/kg bodyweight per day i.v. for two days) was started to delay the development of pulmonary fibrosis [8]. Additionally, we initiated treatment with tamoxifen (3 × 20 mg p.o.), for which a hormone-independent antiproliferative and anti-inflammatory effect is well known in retroperitoneal fibrosis [9]. However, 72 h after paraquat ingestion, desaturation occured, and the patient had to be transferred to the medical intensive care unit. Due to progressive respiratory failure, oxygen supplementation had to be started despite its potentially enhancing effect on paraquat toxicity [3]. On the 9th day after paraquat ingestion, invasive ventilation became necessary due to the progression of pulmonary fibrosis (Figure 2B). Because of the convincing negation of a suicidal attempt, confirmed by repeated psychiatric evaluation, we decided to list the patient for high urgency double lung transplantation. The patient’s condition worsened progressively with development of a systemic inflammatory response syndrome. On his 12th hospital day, FiO2 had to be increased from 50 to 100% due to deteriorated oxygenation, and a veno-venous extracorporeal membrane oxygenation (vvECMO) was implemented (Figure 2C-D). Even with maximum support by vvECMO, multiple drastic declines in gas exchange led to hemodynamic instability, so that the veno-venous system had to be switched to veno-arterial ECMO (vaECMO) on the 20th day. After initial stabilization on a poor clinical level, septic multiorgan dysfunction with kidney, liver and hemodynamic failure developed from the 29th day on. Thirty-two days after paraquat ingestion, the patient died from cardiac failure with pulseless electrical activity. An autopsy was performed, showing fibrotic remodelling of the lungs and ubiquitous signs of shock, such as swelling of the brain, liver and kidneys. Postmortem tissue specimens were examined for paraquat levels (Table 2). For this purpose, frozen tissue specimens were mechanically homogenized, extracted with perchloric acid and neutralized. After pre-purification, paraquat levels in extracts or serum samples were quantified by reverse-phase HPLC and UV detection at 270 nm with a detection limit of 0.01 mg/l for serum and 0.2 μg/g for processed tissues (intraassay imprecision 2.4%).

**Table 1:** Laboratory values after referral (3rd day after paraquat ingestion); pathological values are bold

**Parameter**	**Measured value**	**Standard value**
**Leukocytes**	**17.7 / nl**	4.4 – 11.3 / nl
Erythrocytes	4.65 / fl	4.50 – 5.90 / fl
Hemoglobin	14.5 g/dl	13.5 – 17.5 g/dl
MCV	84.5 fl	80 – 100 fl
MCH	31.2 pg	26 – 34 pg
MCHC	36.9 g/dl	31 – 37 g/dl
INR	1.17	0.9 – 1.25
aPTT	26 sec	26 – 35 sec
s-potassium	3.8 mmol/l	3.6 – 5.4 mmol/l
**s-sodium**	**130 mmol/l**	138 – 148 mmol/l
**s-chloride**	**90 mmol/l**	97 – 108 mmol/l
s-calcium	2.41 mmol/l	2.15 – 2.60 mmol/l
s-phosphate	1.60 mmol/l	0.83 – 1.67 mmol/l
**s-CRP**	**10 mg/l**	< 8 mg/l
**s-creatinine**	**387 μmol/l**	59 – 104 μmol/l
**s-urea**	**18.2 mmol/l**	3.3 – 6.7 mmol/l
s-CK	138 U/l	< 171 U/l
**s-AST**	**697 U/l**	< 35 U/l
**s-ALT**	**1030 U/l**	< 45 U/l
**s-GLDH**	**601 U/l**	< 7 U/l
**s-AP**	**138 U/l**	40 – 129 U/l
**s-γGT**	**441 U/l**	< 55 U/l
s-CHE	6.08 U/l	5.32 – 12.91 U/l
**s-LDH**	**393 U/l**	< 248 U/l
**s-bilirubin**	**75 μmol/l**	< 17 μmol/l

**Table 2:** Paraquat levels in different tissues / materials

**Tissue / material**	**Paraquat level**
Bronchoalveolar lavage	< 0.01 mg/l*
Muscle biopsy	< 0.2 μg/g*
Fat biopsy	< 0.2 μg/g*
**Autopsy tissue specimens**	
Brain	< 0.2 μg/g
Bone marrow	< 0.2 μg/g
Heart	< 0.2 μg/g
Kidney	< 0.2 μg/g
Liver	< 0.2 μg/g
Lung	< 0.2 μg/g
Spleen	< 0.2 μg/g
Thyroid gland	< 0.2 μg/g
Pancreas	< 0.2 μg/g
Bowel	< 0.2 μg/g
Bile	< 0.2 μg/g

*17 d after paraquat ingestion.

Treatment options in paraquat poisoning are limited. This holds especially true for the use of extracorporeal treatment methods, which are currently evaluated in a standardized approach [10]. Since the main problems arise from irreversible lung damage, the major goal is to reduce intestinal absorption and enhance elimination of paraquat before it can be stored in the tissue. Administration of activated charcoal and gastric lavages are regularly performed. However, intestinal absorption of paraquat is a rapid process and high paraquat plasma levels are reached within minutes to 4 hours after paraquat ingestion [11]. When our patient came to the hospital approximately 6 h after paraquat ingestion, serum levels might already have reached their maximum, so charcoal administration and gastric lavage probably had no effect.

Although paraquat is eliminated by the kidneys, forced diuresis has no proven benefit on mortality. The early initiation of charcoal hemoperfusion within the first hours after paraquat poisoning seems to have a beneficial prognostic effect on survival [12], but the majority of patients with paraquat poisoning do not benefit from extracorporeal methods including hemoperfusion, hemodialysis or hemofiltration [13,14]. The reason for that might be that the ingested paraquat reaches the lungs before renal replacement therapy can be implemented. Paraquat is stored in different tissues, expecially the lung, but also in brain, liver, kidney, bile and muscle in varying amounts [15-17], from where it is only released slowly. Finally, immunosuppressive therapy regimens and antioxidants [18] have been used to prevent the development of fibrosis, including high-dose steroids, cyclophosphamid [8], and sirolimus [19], with varying success. But even with this aggressive therapy, pulmonary fibrosis develops in most patients including ours, so that lung transplantation seems to be the last option in absence of contraindications. However, lung transplantation can only be successful, if the body is completely depleted of paraquat. Otherwise, fibrosis would potentially recur in the allograft. The first attempt of single lung transplantation in a case of paraquat poisoning was reported as early as 1968. The transplantation was performed seven days after accidental paraquat ingestion, when there was still paraquat detectable in the patient’s blood (0.4 mg/l) and the explanted left lung (8.50 μg/g). Two weeks after the transplantation, the patient died from respiratory failure. Postmortem histopathology revealed the same paraquat-induced fibrotic changes in both the native right and the transplanted left lung, indicating a recurrence of fibrosis caused by paraquat released from its body stores. At this time, no paraquat could be detected in both the native and the transplanted lung postmortem, but other tissues were not examined [4]. Walder et al. [5] reported the first successful single lung transplantations performed 44 days after paraquat ingestion. The patients showed no signs of recurrent pulmonary fibrosis in the allograft and survived at least one year. However, serum was free from detectable paraquat levels as early as 4 d after paraquat ingestion, when serum levels still reached 0.15 mg/l in our patient despite daily hemodialysis.

Our patient was listed for high-urgency lung transplantation, because all of the described treatment strategies failed. We used extracorporeal support to bridge the time to transplantation, but the patient developed septic multiorgan failure and finally died before a suitable donor organ was available. In postmortem tissue specimen no paraquat could be detected, suggesting that lung transplantation would potentially have been successful. Furthermore, the donation of otherwise undamaged organs would have been possible without endangering the recipient, as has been demonstrated for corneas in single cases [20].

## Conclusions

In a variety of tissue samples obtained postmortem, we can show that complete elimination paraquat after oral ingestion of a lethal dose is achievable. The determined time frame for this complete elimination might be relevant for patients, in which lung transplantation is considered.

## Consent

Written informed consent was obtained from the patient’s legal representative for publication of this Case report and any accompanying images. A copy of the written consent is available for review by the Series Editor of this journal.

## Abbreviations

vvECMO: Veno-venous extracorporeal membrane oxygenation; vaECMO: Veno-arterial extracorporeal membrane oxygenation.

## Competing interests

The authors declare that they have no competing interests.

## Authors’ contributions

JH, MH, JG, GW, CH and JTK were the treating physicians of the patient reported. DG performed autopsy. NKV conducted the measurement of paraquat. AB, SSH, SK and JTK evaluated the test results and designed the manuscript and figures. All of the authors have participated in the discussion and in writing of the submitted manuscript. All authors read and approved the final manuscript.

## Pre-publication history

The pre-publication history for this paper can be accessed here:

http://www.biomedcentral.com/2050-6511/14/45/prepub
